# Effectiveness of Selenium Supplementation in the Treatment of Graves–Basedow Disease: A Scoping Review

**DOI:** 10.3390/medsci13040241

**Published:** 2025-10-24

**Authors:** Hernando Vargas-Uricoechea, Alejandro Castellanos-Pinedo, Karen Urrego-Noguera, María V. Pinzón-Fernández, Ivonne A. Meza-Cabrera, Hernando Vargas-Sierra

**Affiliations:** 1 Metabolic Diseases Study Group, Department of Internal Medicine, Universidad del Cauca, Carrera 6 No. 13N-50, Popayan 190001, Colombia; karenurrego@unicauca.edu.co (K.U.-N.); mpinzon@unicauca.edu.co (M.V.P.-F.); imeza@unicauca.edu.co (I.A.M.-C.); hdvargas@unicauca.edu.co (H.V.-S.); 2 Faculty of Medicine, Universidad del Sinu, Hospital San Jeronimo, Monteria 230001, Colombia; acaspinedo@yahoo.es; 3 Health Research Group, Department of Internal Medicine, Universidad del Cauca, Carrera 6 No. 13N-50, Popayan 190001, Colombia

**Keywords:** Graves–Basedow, selenium, thyroid, autoimmunity, orbitopathy

## Abstract

Background: Graves–Basedow disease (GBD) is an autoimmune thyroid disorder characterized by loss of tolerance to the thyrotropin receptor, with clinical manifestations such as a hyperadrenergic state, goiter, orbitopathy, and myxedema, inter alia. Selenium is a micronutrient, essential for the synthesis of selenoproteins. Selenium deficiency has been linked to an increased risk and exacerbation of GBD and GBD orbitopathy; therefore, it has been suggested that supplementation with this micronutrient could modify some outcomes associated with both conditions. Objectives: The objective of this scoping review was to synthesize and analyze the clinical trials that have evaluated the effectiveness of selenium on different outcomes in patients with GBD or GBD orbitopathy. Methods: The following databases were consulted: PubMed/Medline, Scopus, Biosis, ProQuest, Web of Science, and Google Scholar; and the search terms ‘Graves-Basedow disease’ or ‘Graves’ disease’ or ‘hyperthyroidism’ or ‘Graves’ hyperthyroidism’ or ‘selenium or selenium supplementation’ and ‘effectiveness’ were used. The search was limited to articles published in English between January 2000 and March 2025. To reduce selection bias, each article was reviewed independently by three authors using the Rayyan web tool and the JBI Critical Appraisal Checklist. Results: A total of 15 studies were identified (11 on patients with GBD and 4 on patients with GBD orbitopathy). In GBD, selenium supplementation was associated with significant improvements in TSH, FT4, FT3, TPOAb, TgAb, and TRAb levels; while in GBD orbitopathy, a positive effect of selenium supplementation was found on multiple clinical outcomes. Conclusions: Selenium supplementation in patients with GBD or GBD orbitopathy is associated with favorable biochemical and clinical outcomes.

## 1. Introduction

Autoimmune diseases (AIDs) are a broad group of more than 100 heterogeneous diseases in which the common denominator is the loss of immune tolerance against one or multiple autoantigens. It is estimated that approximately 5% of the population has been diagnosed with an AID, and of these, 34% have been diagnosed with more than one AID. Furthermore, women are more frequently affected than men (almost 80% of all confirmed AIDs diagnoses are made in women) [1,2].

AIDs are generally classified as those that can affect multiple organs or systems [non-organ-specific (NOS)] or those that affect a single organ [organ-specific (OE)]. Among the OE AIDs, the most common is autoimmune thyroid disease (AITD) [3,4].

AITD includes Graves–Basedow disease (GBD) and Hashimoto’s disease (HT), which can exhibit two extremes of clinical presentation, hyperthyroidism (in GBD) and hypothyroidism (in HT) [5].

GBD is characterized by an infiltration of T lymphocytes (TLs) into thyroid tissue, as well as an increase in B lymphocytes (BL) activation and the synthesis and secretion of antibodies (Abs) directed against the thyrotropin (TSH) receptor (TSHR) TRAbs. This results in an autoimmune response that can clinically manifest as a goiter and as hyperthyroidism, ophthalmopathy, and dermopathy, inter alia [6].

GBD has a prevalence of 0.5–2.0%, while that of HT is 5–10%. Multiple associated factors (genetic, epigenetic, environmental, socioeconomic, and nutritional, inter alia) not only influence the pathogenesis, but also the overall frequency of AITD [7,8].

Among the nutritional factors at play, the status of some micronutrients in a given population is considered to affect susceptibility to AITD. Studies in this area have focused on determining the concentrations of micronutrients such as selenium, iodine, iron, zinc, and vitamin D, inter alia, in the blood (whole blood, serum, or plasma), urine (in random or 24 h urine samples), or various tissues [9,10].

In this sense, selenium is an essential micronutrient for the biosynthesis of selenoproteins containing selenocysteine. The thyroid contains the highest amount of selenium per gram of tissue, and most selenoproteins are expressed in the thyroid and participate in the metabolism of thyroid hormones [11,12,13].

Selenium deficiency has been associated with an increased risk of AITD, an effect that could be explained by several mechanisms, such as decreased synthesis and secretion of interferon gamma and other cytokines, accompanied by an alteration in the cellular immune response, along with increased activity of autoreactive TLs and low activity of regulatory TLs (Treg) [14,15,16,17].

The importance of selenium for health lies in its role as a component of selenocysteine (SeCys), which is present in various selenoproteins. SeCys is located in several active enzyme sites that play fundamental roles in the regulation of reactive oxygen species (ROS), energy metabolism, redox status, and the various cellular processes responsible for the innate and adaptive immune responses [16,17,18].

Moreover, among the well-characterized selenoproteins are iodothyronine deiodinases (DIOs), glutathione peroxidases (GPXs), and thioredoxin reductases (TXNRDs), all of which are enzymes involved in thyroid hormone metabolism, the regulation of redox states, and protection from oxidative damage [19,20].

Therefore, selenium deficiency can result in a reduction in the expression and activity of these enzymes, resulting in increased T4 levels and decreased T3 levels [20,21].

Additionally, selenium deficiency (corresponding to a serum concentration < 70 µg/L) has also been documented as being associated with AITD; in fact, multiple studies have shown that selenium supplementation in areas deficient in this micronutrient decreases thyroid Ab concentrations, suggesting that it could modify the natural course of AITD [22,23].

Several studies have documented that serum selenium concentrations are significantly lower in individuals with GBD (compared to healthy individuals), resulting in selenium deficiency being considered a risk factor for the development of GBD [22,23,24,25].

This has, in turn, led to the suggestion that selenium supplementation could have a favorable effect not only in terms of the prevention of AITD (and, in this sense, GBD), but also in terms of different biochemical outcomes (e.g., TSH, FT4, FT4 concentrations, and thyroid Ab titers) or clinical outcomes associated with the disease (e.g., signs and symptoms of hyperthyroidism or ocular findings—GBD orbitopathy) [23,24,25].

These concepts then led to the design and delivery of clinical studies that evaluated the effectiveness of selenium supplementation in patients with GBD, with or without ophthalmopathy. However, despite the evidence from these clinical studies, there is still no universally accepted criterion for the use of selenium in such patients.

The objective of this scoping review is to evaluate the effectiveness of the use of selenium in patients with GBD (or with GBD orbitopathy) who have undergone the usual treatment for the disease (or who have previously been treated) in relation to a range of clinical and/or biochemical outcomes.

## 2. Materials and Methods

### 2.1. Literature Search and Selection Criteria

Using a modified version of the Population, Interventions, Comparators, and Outcomes (PICO) framework, we formulated the research question and defined the eligibility criteria for the scoping review (Table 1).

Subsequently, a structured literature search was carried out in PubMed/Medline, Scopus, Biosis, ProQuest, Web of Science, and Google Scholar for articles published from January 2000 to March 2025 (human trials, clinical trials, meta-analyses, reviews, scoping reviews, and systematic reviews).

The following search terms were used: ‘Graves-Basedow disease’ or ‘Graves’ disease’ or ‘hyperthyroidism’ or ‘Graves’ hyperthyroidism’ or ‘selenium or selenium supplementation’ and ‘effectiveness.’

The search strategy was as follows: Graves-Basedow disease [Title/Abstract] OR Graves’ disease [Title/Abstract] OR hyperthyroidism [Title/Abstract] OR Graves’ hyperthyroidism [Title/Abstract] OR Selenium intake [Title/Abstract] OR Selenium supplementation AND Effectiveness [Title/Abstract] OR Outcomes [Title/Abstract].

**Figure 1:**
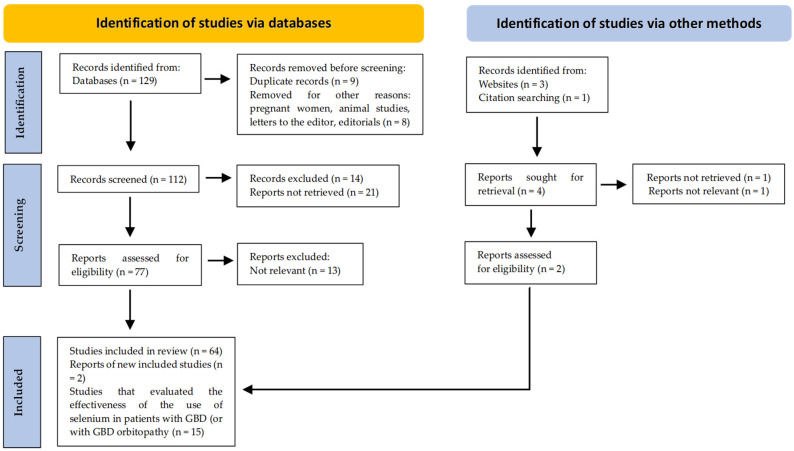
PRISMA flow diagram. Method for the selection of articles.

Following the Preferred Reporting Items for Systematic Reviews and Meta-Analyses (PRISMA) guidelines, a total of 15 studies were included: 11 on individuals with GBD and 4 on patients with GBD orbitopathy (Figure 1).

### 2.2. Data Extraction

The titles and abstracts of all the studies were independently reviewed by three investigators (H.V.-U., A.C.-P., and H.V.-S) using the Rayyan web tool (this further helped reduce selection bias). Full texts of the studies that met the initial inclusion criteria were obtained and reviewed, and the data were extracted through a standardized template, using a predefined data form created in Excel.

In the presence of discrepancies in data extraction, the investigators collaboratively conducted a second round of analysis and extraction to validate the information obtained. Each article was scrutinized according to the JBI Critical Appraisal Checklist; only articles written in English were considered.

The eligible studies and the inclusion and exclusion criteria (according to the defined categories) are described in Table 2.

### 2.3. Data Analysis

The following data were collected in the review: design type, country, inclusion criteria, interventions, selenium dose, number of participants, follow-up time, and clinical and/or biochemical outcomes.

The heterogeneity found in the 15 studies, in relation to aspects such as the definition of the disease, previous or current treatment of hyperthyroidism, clinical and/or biochemical outcomes, and follow-up time, did not allow for a statistical analysis or meta-analysis. Consequently, we conducted a descriptive analysis, using a narrative approach, to summarize and synthesize the most representative findings of the selected studies.

This scoping review was registered on the “International Platform of Registered Systematic Review and Meta–Analysis Protocols INPLASY” (Registration number: INPLASY202580095; DOI: 10.37766/inplasy2025.8.0095) and conducted according to the “Reporting Checklist for Systematic Reviews Based on the PRISMA guidelines” (Table S1).

## 3. Results

### 3.1. General Characteristics of the Studies and Participants

A total of 15 clinical trials were identified, 11 of which were developed with individuals with GBD [26,27,28,29,30,31,32,33,34,35,36] and 4 with patients with GBD orbitopathy [37,38,39,40]. Table 3 and Table 4 summarize the design of the clinical trials, the baseline statuses and selenium doses used, and the follow-up times and outcomes for participants diagnosed with GBD (or GBD orbitopathy) and assigned to receive selenium management.

These studies are generally notable for their small samples, with follow-ups ranging from the very short (e.g., 4 weeks) up to the very long (e.g., 5 years). The majority of the population evaluated consisted of women, with mean ages of 39.1 years in the intervention group (with selenium) and 39.7 years in the control group (in the 11 studies of participants with GBD), and 42.2 years in the intervention group and 44.6 years in the control group (in the 4 studies of participants with GBD orbitopathy).

### 3.2. Basal Selenium Concentrations and Doses Used in the Studies

Baseline selenium concentrations were assessed in 4 of the 11 studies of participants with GBD (normal concentrations were observed in 3 studies, and a low concentration was observed in 1); of the 4 studies of individuals with GBD orbitopathy, selenium concentrations were assessed in 2 (normal concentrations were observed in both studies, but in 1 study, concentrations were measured only in the intervention group).

The selenium doses used ranged from 60 to 300 µg/day; various types of selenium (selenite, selenium yeast, selenium glycinate, selenomethionine, selenomethionine + selenium yeast, L-selenomethionine, capsules of antioxidants, selenious yeast, and selenium glycinate) were used.

### 3.3. Concomitant or Previously Used Management for GBD

In 10 of the 11 studies on participants with GBD, methimazole was used as a baseline treatment. In 1 of these, baseline management was carried out with antioxidants. In contrast, in the studies concerning GBD orbitopathy, patients were previously managed with methimazole, radioactive iodine, or thyroidectomy (and remained euthyroid throughout the studies).

### 3.4. Severity of GBD Orbitopathy

In the studies on patients with GBD orbitopathy, participants were classified as follows: mild orbitopathy and euthyroidism (one study); mild and active orbitopathy (one study); moderate-to-severe inactive orbitopathy (one study); and mild-to-moderate orbitopathy (one study).

### 3.5. Clinical and Biochemical Outcomes (Before and After Selenium Intervention) for Participants with GBD or GBD Orbitopathy

The studies concerning the participants with GBD primarily assessed (before and after selenium intervention) outcomes such as TSH, FT4, FT3, TPOAb, TgAb, and TRAb concentrations; remission and recurrence rates of hyperthyroidism; and clinical and/or biochemical control of hyperthyroidism.

Meanwhile, in the studies of individuals with GBD orbitopathy, the effects of selenium treatment on specific outcomes—such as Clinical Activity Score (CAS), improvement in total GBD orbitopathy-related Quality of Life (GO–QOL), visual functioning score (GO–QOL change), psychological functioning score (GO–QOL), palpebral (eyelid) aperture change, improvement in palpebral (eyelid) aperture, exophthalmos change, and improvement in exophthalmos—were measured.

In 9 of the 11 studies concerning individuals with GBD, and in all 4 studies on individuals with GBD orbitopathy, at least one significant and favorable outcome was found in the group of participants who received selenium (Table 5).

### 3.6. Favorable Biochemical Outcomes After Selenium Intervention for Participants with GBD

In summary, 9 of the 11 studies (on patients with GBD) in which at least one significant and favorable result (biochemical outcomes) was achieved with the use of selenium found the following:

One study demonstrated that a euthyroid state had been significantly achieved [26]; one demonstrated a significant reduction in FT4 levels [27]; one demonstrated a significant increase in TSH levels, with a decrease in FT4 levels [28]; one demonstrated a significant increase in TSH levels, with a decrease in FT3, FT4, TgAb, and TPOAb and TRAb titers [29]; one demonstrated an increase in the effect of antithyroid drugs in patients with recurrent GBD [30]; one demonstrated a reduction in TPOAb and TRAb titers and a reduction in the incidence of hypothyroidism [33]; one demonstrated a significant decrease in TRAb levels [34]; one demonstrated a decrease in FT3 and FT4 concentrations as well as TRAb, TPOAb, and TgAb titers [35]; and, one study demonstrated that the use of selenium enhances the effect of antithyroid drugs (when selenium and vitamin D levels are suboptimal) [36].

### 3.7. Favorable Clinical Outcomes After Selenium Intervention for Participants with GBD Orbitopathy

Furthermore, all four studies on patients with GBD orbitopathy showed at least two clinical outcomes in favor of the use of selenium [37,38,39,40]. The outcomes can be summarized as follows: CAS change at 6 months (three of four studies were in favor) and 12 months (two of two studies were in favor); improvement in total GO–QOL at 6 months (one of two studies in favor) and 12 months (one of two studies in favor); visual functioning score GO–QOL change at 6 months (one of three studies in favor) and/or 12 months (one of two studies was in favor); psychological functioning score GO–QOL change at 6 months (one of three studies in favor), and 12 months (one of two studies in favor); palpebral (eyelid) aperture change at 6 months (zero of two studies in favor); improvement in palpebral (eyelid) aperture at 6 months (one of two studies in favor); exophthalmos change at 6 months (zero of two studies in favor); and improvement in exophthalmos at 6 months (zero of two studies in favor).

## 4. Discussion

In this scoping review, we found that 9 of the 11 studies on patients with GBD and all 4 studies on patients with GBD orbitopathy demonstrated selenium use had a significant benefit.

Among patients with GBD, the benefits were achieved in six clinical scenarios (hyperthyroidism due to GBD; GBD treated with methimazole; newly diagnosed GBD; untreated hyperthyroid patients with GBD; recurring GBD; and patients with GBD after radioactive iodine treatment), with favorable outcomes in relation to increased TSH levels and decreased FT4, FT3, TPOAb, TgAb, and TRAb levels.

Meanwhile, in patients with GBD orbitopathy, benefits were achieved in four major clinical scenarios (mild GBD orbitopathy with euthyroidism; mild and active GBD orbitopathy (CAS > 3); inactive moderate-to-severe GBD orbitopathy; and mild-to-moderate GBD orbitopathy), with favorable outcomes in relation to quality of life, reduced ocular involvement, and slowed progression of the disease (in mild Graves’ orbitopathy); differences in palpebral fissure and CAS and eyelid aperture (even in inactive moderate-to-severe GBD orbitopathy); and the early course of mild-to-moderate GBD orbitopathy.

Trying to explain the beneficial effect of selenium on GBD and GBD orbitopathy can be difficult, given the biological and molecular complexity of AITD. GBD originates from the loss of host tolerance toward the TSHR, and TL activation induces the synthesis and secretion of inflammatory cytokines and the release of chemokines by thyroid follicular cells, generating an amplified inflammatory response, with an increase in the synthesis and secretion of TRAb [3,5,41].

Consequently, direct stimulation of TRAbs on the TSHR induces inappropriately high secretion of thyroid hormones, goiter, and extrathyroidal manifestations (especially orbitopathy and/or myxedema) [42].

The close interaction between genetic, non-genetic, epigenetic, and environmental factors influences the risk of developing GBD. In this context, among environmental factors, selenium is one of the most studied in relation to the association between deficiency and an increased risk of AITD (particularly with GBD and GBD orbitopathy) [16,42].

Therefore, the prevalence of selenium deficiency varies (depending on the geographic area studied), and some studies have suggested that the differences found in the effectiveness of selenium supplementation in patients with GBD and/or GBD orbitopathy could only be reflected in individuals with a deficiency in this micronutrient [16,43,44].

Furthermore, it should be noted that selenium intake from organic sources (e.g., selenomethionine and selenocysteine) is associated with greater absorption and bioavailability than that from inorganic sources (e.g., selenite and selenate), and that inorganic forms are retained in the body to a lesser extent than selenomethionine and selenocysteine [12,15,18,21]. This aspect related to absorption, bioavailability, and the source of selenium could also explain (at least in part) the findings of this review (and the variability of these results).

However, this scoping review describes the results in favor of selenium supplementation for people with GBD or GBD orbitopathy (in 13 of the 15 selected studies), taking into account that only 6 of the studies determined the baseline selenium status. Thus, the results suggest that, regardless of baseline selenium status, selenium supplementation (associated with baseline management with methimazole) has a positive effect on various biochemical outcomes (in GBD) and on multiple clinical and biochemical outcomes (in GBD orbitopathy).

Intriguingly, in one of the studies of mild-to-moderate GBD orbitopathy, a benefit of selenium use was found in study participants in the short term, but not in the long-term outcomes (which may be an effect of the natural evolution of GBD orbitopathy, where a decrease in its activity over time is observed) [22,23,40].

The above can be explained by the fact that selenium supplementation can induce an immunomodulatory effect in patients with AITD; for example, in a mouse model of autoimmune thyroiditis, selenium was observed to decrease lymphocytic infiltration in the thyroid, with the upregulation of Treg and the expression of GPX and TXNRD [45].

Other studies have found that selenoprotein deficiency can decrease calcium influx during the activation of various cells involved in the immune response, affecting the ability to respond to host and foreign antigens [46]. It has also been proposed that selenium may inhibit cell proliferation and the secretion of several proinflammatory cytokines (e.g., IFN-γ and TNF-α) [14,15,42,43].

However, most of the evidence for selenium’s potential immunomodulatory effect on GBD and GBD orbitopathy comes from animal models. Therefore, information from RCTs is quite scarce and limited, with studies varying considerably in terms of study design, methods for assessing immune function and response, selenium dose used, and follow-up time, among other factors.

**Figure 2:**
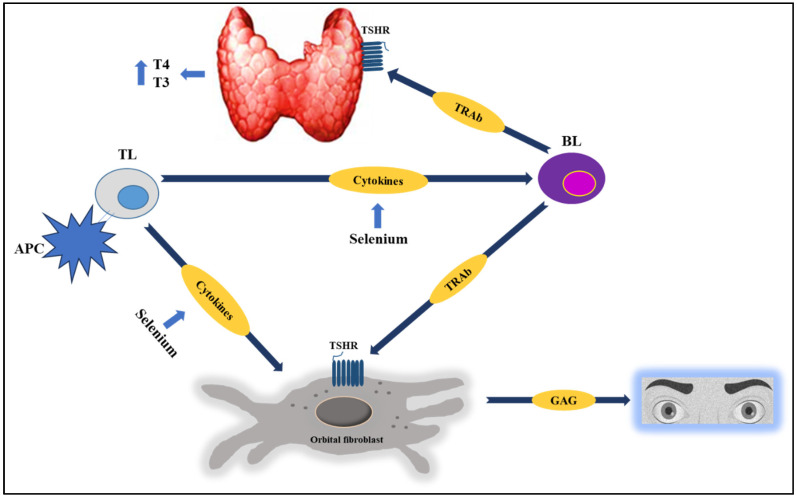
In AITD, selenium has a potential immunomodulatory effect, as it is capable of inhibiting the synthesis of inflammatory cytokines, attenuating TL infiltration into the thyroid, and reducing thyroid Abs (TRAb) levels, among other effects. Studies suggest that selenium supplementation (along with basic management, especially with methimazole) favorably modifies multiple clinical and/or biochemical outcomes in patients with GBD or GBD orbitopathy. See the text for further details. Abbreviations: TSHR: Thyrotropin receptor; BL: B lymphocyte; TL: T lymphocyte; APC: antigen-presenting cell; GAG: glycosaminoglycans.

Several studies suggest that the effect of selenium supplementation on humoral immunity is of low impact and magnitude [9,43,44]. However, this review found that supplementation has a beneficial effect on the levels of different thyroid Abs, while the effect on the cellular immune response is less clear (and among the RCTs selected in this review, none directly evaluated this aspect) Figure 2.

On the other hand, when selenium intake is adequate, the intracellular glutathione peroxidase and thioredoxin peroxidase systems protect thyrocytes from these peroxides. Additionally, selenium deficiency produces a significant reduction in the activity of the selenoprotein glutathione peroxidase, which removes H_2_O_2_ (promoting lipid peroxidation). Selenoproteins also prevent the excessive formation of ROS, which promote chronic inflammation and autoimmunity, as they are capable of regulating the effector function of TLs [10,11,42,43].

Consequently, adequate selenium intake could promote an effective immune response toward Th1 lymphocytes, thus avoiding a “switch” to a Th2 lymphocyte-mediated response (one of the hallmarks of GBD). In fact, some studies suggest that selenium supplementation may attenuate the Th2-mediated immune response, inducing a predominantly Th1-mediated response [6,10,42].

## 5. Strengths and Weaknesses

This scoping review has several limitations, such as the number of RCTs with small sample sizes, the widely varying follow-ups of participants, the heterogeneity of the inclusion criteria, and the lack of clear descriptions of the methods of diagnosis, disease severity, and baseline selenium statuses. Furthermore, the outcomes assessed were not standardized.

The use of other ATDs (propylthiouracil or carbimazole) and concomitant therapies (such as cholestyramine) was not taken into account either. It should also be noted that none of the studies assessed the basal statuses of other micronutrients, which may influence selenium metabolism and basal status (e.g., iodine, vitamin D, zinc, iron, etc.). Additionally, some studies did not provide sufficient information on and/or the clinical and biochemical characteristics of participants.

Therefore, conclusions based on our findings should only be extrapolated to patients with conditions similar to those of the participants in the selected studies.

However, this review has some strengths—for example, the rigorous process carried out in the search for information and current evidence on the subject, and the collection and selection of studies from the described databases (PubMed/Medline, Scopus, Biosis, ProQuest, Web of Science, and Google Scholar)—which allowed us to establish in a broad and precise manner the current evidence of the effectiveness of selenium in terms of its various clinical and/or biochemical outcomes for patients with GBD and GBD orbitopathy.

## 6. Future Implications

Finally, there is an urgent need to develop RCTs with more robust designs and that involve humans, evaluating the mechanisms by which selenium supplementation could affect the different clinical, biochemical, metabolic, and/or imaging outcomes in patients with GBD and with GBD orbitopathy, at different stages of severity and activity of the disease, as well as the doses to be administered, the duration of treatment, and the effect on the risk of relapse and clinical progression.

It should also be evaluated and clarified in this type of study (RCT) whether the basal selenium status determines the success of selenium supplementation in patients with GBD and/or GBD orbitopathy, especially given the fact that the only two studies that did not show a benefit from such supplementation were those that evaluated the basal selenium status prior to the intervention (and in which its serum concentration was normal). This concept can also be extrapolated to the basal status of other micronutrients involved in a greater susceptibility to the presence of AITD.

Furthermore, because few studies measured selenium levels at baseline or follow-up, it is unknown whether participants in the studies assessed in this review may have had elevated serum selenium concentrations (and whether the doses used were sufficient or supraphysiological); therefore, future studies should focus on maintaining normal serum selenium concentrations (63–159 μg/L) during follow-up, since supraphysiological doses have been associated with outcomes such as hair loss, nausea, vomiting, dermatitis, different types of cancer, and increased mortality [24,26,47].

Additionally, the concomitant use of selenium should also be considered in patients with GBD orbitopathy who are receiving other non-surgical treatments (glucocorticoids, azathioprine, rituximab, mycophenolate mofetil, teprotumumab, tocilizumab, and orbital radiotherapy, inter alia), specifically to determine whether the use of selenium results in a synergistic effect or decreases the need for more aggressive treatments (orbital radiotherapy or surgical management) [48,49].

## 7. Conclusions

In patients with GBD, selenium supplementation combined with methimazole therapy is significantly associated with an increase in TSH levels and a reduction in FT4, FT3, TPOAb, TgAb, and TRAb levels. These findings were consistent across six clinical scenarios (hyperthyroidism due to GBD, GBD treated with methimazole, newly diagnosed GBD, untreated hyperthyroid patients with GBD, recurring GBD, and patients with GBD after radioactive iodine treatment).

For patients with GBD orbitopathy, selenium supplementation is significantly associated with multiple clinical outcomes (improved quality of life, reduced ocular involvement, and slowed progression of the disease; differences in palpebral fissure, CAS, and eyelid aperture; and an improved early course of GBD orbitopathy), specifically in four clinically possible scenarios (mild GBD orbitopathy with euthyroidism; mild and active GBD orbitopathy; inactive moderate-to-severe GBD orbitopathy; and mild-to-moderate GBD orbitopathy).

## Figures and Tables

**Table 1:** Inclusion criteria adopted in the scoping review.

PICO Elements	Inclusion Criteria
Population	Patients diagnosed with Graves-Basedow disease (GBD) or GBD orbitopathy
Intervention	Supplementation with different forms of oral selenium
Comparison	ATD or placebo or other interventions
Outcome	- Clinical and/or biochemical control of hyperthyroidism - Clinical control of GBD orbitopathy

Abbreviations: ATD: antithyroid drugs; PICO: Population, Interventions, Comparators, and Outcomes framework.

**Table 2:** Categories, inclusion, and exclusion criteria of eligible studies.

Categories	Inclusion Criteria	Exclusion Criteria
Topic	Studies correspond to selenium supplementation, and/or Graves-Basedow disease (GBD) or GBD orbitopathy	Not applicable
Selection of databases	PubMed/Medline, Scopus, Biosis, ProQuest, Web of Science, and Google Scholar	Different databases
Search limits for studies (according to time interval)	From January 2000 to March 2025	Not applicable
Population/Target group	Humans/Adults	Other types of studies, for example, in pregnant women or animals
Context	Any geographic area, continent, or country	Not applicable
Study design	Clinical trials, meta–analyses, reviews, scoping reviews, and systematic reviews	Letters, commentaries, preprints, letters to the editor, non–peer–reviewed articles and conference abstracts
Data extraction	Standardized template using a predefined data form (in Excel)	Other forms of data extraction
Language	English	Others

**Table 3:** Clinical trial design and general characteristics, follow-up time, and outcomes of participants diagnosed with GBD, assigned to receive selenium treatment.

Author; Country; Year of Publication and [ref.]	Study Design	Inclusion Criteria	Selenium Status at Baseline	Interventions and Selenium Dose	Participants (n)		Mean Age (Years, SD)		Female (%)		**Follow-Up Time**	**Outcome**
					Selenium	Control	Selenium	Control	Selenium	Control		
Bacic Vrca V; Croatia; 2004 [26]	Randomized, placebo-controlled trial	Patients with GBD treated with methimazole	Not described	Methimazole 120 mg daily for the first week, 80 mg daily the second week, 60 mg daily the third and fourth week, 40 mg daily for the following 4 weeks + capsule of antioxidants (vitamins C and E, beta-carotene, including 60 µg of selenium) or methimazole (in the same dose previously noted)	27	28	44 ± 12	41 ± 14	86	96	30 and 60 days	Patients receiving antioxidant supplementation plus methimazole achieved a euthyroid state more quickly than those on methimazole monotherapy
Lai J; China; 2014 [27]	Randomized clinical trial	Patients with hyperthyroidism due to GBD	Was not evaluated	Methimazole 5–30 mg/day + selenious yeast (100 µg/day) or methimazole 5–30 mg/day	60	60	40 (mean)	40 (mean)	77	77	3 months	In the selenium intervention group, a significant reduction in FT4 levels was observed
Calissendorff J; Sweden; 2015 [28]	Randomized, double-blind, placebo-controlled trial	Patients with newly diagnosed GBD	Selenoprotein P concentration was determined	Treatment with antithyroid drugs was given with methimazole (15 mg/twice a day) and levothyroxine. The patients were randomized to treatment with 200 µg selenium/day as yeast tablets or to placebo	19	19	35 (range: 19–49)	44 (range: 23–55)	79	84	6, 18, and 36 weeks	Reduction of FT4 after 18 and 36 weeks and an increase in TSH after 18 weeks in the selenium-supplemented group. The median concentration of selenoprotein P rose in the treatment group (with selenium)
Gong M; China; 2015 [29]	Randomized clinical trial	Patients with hyperthyroidism due to GBD	Was not evaluated	Methimazole 15–30 mg/day + selenious yeast (200 µg/day) or methimazole 15–30 mg/day	40	40	36 (mean)	36 (mean)	56	56	6 months	In the selenium intervention group, a significant reduction in FT3, FT4, TgAb, TPOAb, and TRAb levels and a significant increase in TSH levels were observed
Wang L; China; 2016 [30]	Prospective pilot, quasi-random study	Recurrent GBD (history of hyperthyroidism remission after a finished regular regimen with antithyroid drugs)	Was not evaluated	All patients received the routine treatment using methimazole, while patients allocated to the selenium group (sodium selenite; 200 µg/day). received additional selenium therapy for 6 months.	21	20	37.4 ± 15	38.9 ± 14.3	76.2	90	18 months	Selenium supplementation can enhance the effect of antithyroid drugs in patients with recurrent GBD
Kahaly GJ; Germany; 2017 [31]	Randomized, double-blind, placebo-controlled trial	Untreated hyperthyroid patients with GBD	Normal values	In addition to methimazole, patients received for 24 weeks either sodium selenite 300 µg/day or placebo	35	35	44.5 ± 13.8	44.5 ± 13.4	80	74.3	24 and 36 weeks	Supplemental selenium did not positively affect the clinical course and the serological parameters of selenium-sufficient, hyperthyroid patients with GBD
Leo M; Italy; 2017 [32]	Randomized clinical trial	Untreated hyperthyroidism due to GBD	Normal values	Methimazole or methimazole plus selenium (L- selenomethionine, 166 µg/day)	15	15	43 ± 11	38 ± 11	93	87	90 days	Selenium supplementation does not offer any advantage in terms of short-term control of hyperthyroidism if selenium intake is adequate. Selenium is likely useful if the patient is selenium-deficient
Hui T; China; 2017 [33]	Randomized clinical trial	Participants with GBD after radioactive iodine treatment	Was not evaluated	Methimazole 20 mg/day + selenious yeast (200 µg/day) or methimazole 20 mg/day	121	120	28 (mean)	28 (mean)	75	75	9 months	The use of selenium yeast after radioactive iodine treatment of GBD reduces the titers of TPOAb and TRAb and reduces the incidence of hypothyroidism
Huan F; China; 2017 [34]	Randomized clinical trial	Participants with GBD	Was not evaluated	Methimazole 15–30 mg/day + selenious yeast (300 µg/day) or methimazole 15–30 mg/day	30	30	38 (mean)	38 (mean)	58	58	6	Selenium supplementation significantly reduced TRAb levels in the group receiving selenium supplementation
Xu B; China; 2019 [35]	Randomized clinical trial	Newly diagnosed patients with hyperthyroidism due to GBD	Was not evaluated	Methimazole or methimazole plus selenium (300 µg/day)	44	50	38.9 ± 11.6	40.2 ± 12.6	68	62	6 months	The group that received methimazole plus selenium had lower levels of FT3 and FT4 and lower TRAb, TPOAb, and TgAb expressions than the methimazole group
Gallo D; Italy; 2022 [36]	Randomized, single-blinded, controlled, intervention trial	Patients with newly onset GBD and marginal/insufficient selenium and vitamin D levels	Serum selenium concentration <120 µg/L (borderline low levels)	Methimazole or methimazole plus selenium 100 µg/day (selenomethionine 83 µg + selenium yeast 17 µg and cholecalciferol 7000 IU weekly	21	21	45.8 ± 9.3	47.7 ± 11.4	81	95	270 days	Reaching optimal selenium and vitamin D levels increases the early efficacy of methimazole treatment when selenium and vitamin D levels are suboptimal

**Table 4:** Clinical trial design and general characteristics, follow-up time, and outcomes of participants diagnosed with GBD orbitopathy, assigned to receive selenium treatment.

Author; Country; Year of Publication and [ref.]	Study Design	Inclusion Criteria	Selenium Status at Baseline	Interventions and Selenium Dose	Participants (n)		Mean Age (Years, SD)		Female (%)		**Follow-Up Time**	**Outcome**
					Selenium	Control	Selenium	Control	Selenium	Control		
Marcocci C; H; Netherlands; 2011 [37]	Randomized, double-blind, placebo-controlled trial	Patients with mild GBD orbitopathy, with euthyroidism, as a result of management with antithyroid drugs, radioactive iodine, or thyroidectomy	Was not evaluated	Selenite (100 μg twice daily); pentoxifylline (600 mg twice daily); or placebo (twice daily)	54	48 (pentoxifylline); 50 (placebo)	43 ± 11	43.7 ± 12.4 (pentoxifylline); 44.6 ± 10.7 (placebo)	89	82	12 months	Selenium administration significantly improved the quality of life, reduced ocular involvement, and slowed progression of the disease in patients with mild Graves’ orbitopathy
Almanza-Monterrubio M; Mexico; 2021 [38]	Controlled, randomized, single-center trial	Patients with mild and active GBD orbitopathy by CAS > 3.	Was not evaluated	Selenium tablets (100 µg twice/day) or placebo (100 μg of starch twice a day)	15	15	40.7 ± 10.5	42.5 ± 11.8	80	73	6 months	Significant differences in palpebral fissure and CAS between the pretreatment values and six months after treatment in the selenium group
Potita P; Thailand; 2024 [39]	Single-center, placebo-controlled, double-masked, randomized trial	Inactive moderate–to–severe GBD orbitopathy Patients. All participants were euthyroid for at least 6 months before study entry.	Normal values	200 μg/day of selenium glycinate capsule vs. placebo	13	12	42.2 ± 12.7	48.1 ± 1.6	46	75	6 months	Selenium supplementation has a positive effect on eyelid aperture even in inactive moderate-to-severe GBD orbitopathy patients with a sufficient baseline selenium level
Wang C; China; 2024 [40]	Prospective controlled cohort clinical trial	Patients with mild-to-moderate GBD orbitopathy.	Selenium concentration was only determined in the intervention group at baseline (77.51 μg/L ± 16.23), but not in the control group.	Selenium yeast (100 μg twice/day) vs. placebo. Duration of intake: 6 months	46	28	42.8 ± 12.22	44.1 ± 12.7	65	61	5 years	Six months of selenium supplementation may effectively change the early course of mild-to-moderate GBD orbitopathy, but this regimen makes no difference in long-term outcomes

**Table 5:** Number of studies showing favorable results with selenium intervention, according to biochemical and/or clinical findings.

Outcomes Index	Number of Studies Included in the Review (GBD, n = 11)	Number of Studies Included in the Review (GBD Orbitopathy, n = 4)
Number (n) of studies that evaluated (≥2 biochemical outcomes) such as: TSH, FT4, FT3, TRAb, TPOAb, TgAb, inter alia	11	Not applicable
Number (n) of studies that evaluated (≥2 clinical outcomes) such as: CAS, GO–QOL, ocular symptoms and signs, inter alia	Not applicable	4
Number (n) of studies of participants with GBD, where ≥1 significant and favorable result (biochemical outcomes) was found	9	Not applicable
Number (n) of studies of participants with GBD orbitopathy, where ≥1 significant and favorable result was found	Not applicable	4

Abbreviations: CAS: Clinical Activity Score; FT3: free triiodothyronine; FT4: free thyroxin; GBD: Graves–Basedow disease; GO–QOL: Graves’ Orbitopathy Quality of Life; TgAb: antibodies directed against the thyroglobulin; TPOAb: antibodies directed against the thyroid peroxidase; TRAb: antibodies directed against the thyrotropin receptor; TSH: thyrotropin.

## Data Availability

No new data were created or analyzed in this study.

## Supplementary Materials

The following supporting information can be downloaded at: https://www.mdpi.com/article/10.3390/medsci13040241/s1, Table S1: PRISMA 2020 Main Checklist.
